# Masking terminal neo-epitopes of linear peptides through glycosylation favours immune responses towards core epitopes producing parental protein bound antibodies

**DOI:** 10.1038/s41598-020-75754-7

**Published:** 2020-10-28

**Authors:** Robert Pon, Anne Marcil, Wangxue Chen, Christine Gadoury, Dean Williams, Kenneth Chan, Hongyan Zhou, Amalia Ponce, Eric Paquet, Komal Gurnani, Anindita Chattopadhyay, Wei Zou

**Affiliations:** 1 Human Health Therapeutics Research Center, National Research Council of Canada, 100 Sussex Drive, Ottawa, ON K1A 0R6 Canada; 2 Digital Technology Research Center, National Research Council of Canada, 1200 Montreal Road, Ottawa, ON K1A 0R6 Canada

**Keywords:** Biological techniques, Immunology, Chemistry

## Abstract

Glycosylation of hydrophobic peptides at one terminus effectively increases their water-solubility, and conjugation through the opposing end to a carrier protein, renders them more immunogenic. Moreover, the glycosylation minimizes antibody responses to potentially deleterious, non-productive terminal neo-epitope regions of the peptides, and consequently shifts peptide immunogenicity towards the core amino acid residues. As proof of concept, glycopeptide-protein conjugates related to influenza hemagglutinin (HA), neuraminidase (NA), and the dimerization loop region of human epidermal growth factor receptor 2 (Her2), demonstrated a favorable production of core peptide specific antibodies as determined by ELISA studies. Furthermore, glycosylated Her2 peptide conjugate antisera were also shown to recognize full length Her2 protein by ELISA and at the cell surface through flow cytometry analysis. In contrast, unmasked peptide conjugates generated significant antibody populations that were specific to the terminal neo-epitope of the peptide immunogen that are notably absent in parental proteins. Antibodies generated in this manner to peptides in the dimerization loop of Her2 are also functional as demonstrated by the growth inhibition of Her2 expressing SKBR3 carcinoma cells. This method provides a technique to tailor-make epitope-specific antibodies that may facilitate vaccine, therapeutic and diagnostic antibody development.

## Introduction

The efficacy of a vaccine often depends on the production of functional antibodies that protect the host from bacterial and viral infections or inhibit cancer development. However, it has been observed that certain non-functional antibodies raised by seasonal flu vaccines may worsen influenza virus infection from related but different viral strains^1^; and non-functional antibodies to protein-based cancer vaccines have been associated with deleterious cancer growth^2^. In this context, it is desirable to formulate a vaccine that only includes key immunogenic epitopes in order to selectively produce desired antibodies; a goal that has so far remained elusive. Targeted specific responses are one of the strengths of monoclonal antibody (mAb) therapy, and most mAb therapeutics recognize conformational antigen epitopes. This in itself poses significant challenges such as to the identification or mapping of these conformational epitopes^3^, or to use reverse engineering strategies to construct efficacious B-cell epitope vaccines^4,5^. Additionally, higher order (secondary, tertiary and quaternary) structures can be easily disrupted by mutations leading to loss of antigenicity, resulting in outcomes such as the annual need to reformulate seasonal flu vaccines and the decreased effectiveness of anti-cancer mAbs over time. A sensible alternative is to develop effective peptide-based vaccines, triggering a protective immune response where the antigenicity of the flexible target epitope is expected to be maintained unless mutations occur specifically within the targeted peptide sequence. In addition, peptide conjugate vaccines also allows for targeting conserved regions of proteins that are often dwarfed by immune responses to more immunodominant regions, such as highly variable conformational epitopes. Along these same lines, the development of mAbs specific to conserved epitopes such as influenza hemagglutinin stem regions, can be valuable diagnostic and lot release tools. Taken together, specific peptide targeting strategies would be advantageous for both vaccine formulation and mAb production for therapeutics and biological tool development.

Hydrophilic peptides and N- or C-terminal region sequences are more immunogenic relative to hydrophobic and core region peptides^6,7^. In a manner analogous to hapten-protein conjugates, the antibody response to peptide-protein conjugates often results in antibody production with a bias towards pocket-type binding of the peptide termini; most likely attributable to the initiating interaction of B-cell receptors with the exposed sequences of the peptide-protein conjugate. For example, most antibodies raised to variable lengths of HA peptide (4–10 amino acids)-KLH conjugates were found to bind only the four terminal amino acids^8^. In other words, the terminal peptide neo-epitope may be immunodominant leading to the production of antibodies that do not cross-react with the neo-epitope lacking parent protein. This phenomenon can be exacerbated when the peptide antigen is also a poorly immunogenic self-antigen (e.g. a cancer antigen) as opposed to foreign antigens such as those from microbial pathogens. Thus potential immunodominant responses towards neo-epitope peptide termini, along with conformational mismatches with parent protein, may explain why peptide conjugates often fail to raise adequate protein-binding antibodies. This explanation is not sufficient alone however, since it has been reported that protein-reactive antibodies could be raised by short peptides^9^. Since it is known that protein glycosylation can mask B-cell epitopes^10^, we reasoned that masking the peptide termini will decrease its relevance as an immunodominant determinant of peptide conjugate vaccines, resulting in an immune response bias to the core region sequences, which are shared in common with its parental protein.

Masking linear peptide termini can be accomplished by its fusion to multiple peptides or protein, which may have the added benefit of increased peptide immunogenicity, but also runs the risk of perturbations in antibody specificity. For example, antibodies raised to a fusion peptide containing the N-terminal 1–13 amino acids of HA2 (a conserved epitope of influenza hemagglutinin) failed to generate specific antibodies against this sequence^11^. Similarly, anti-matrix protein 2 (M2) and nuclear protein (NP) peptides fused with additional T-cell epitopes as a potential influenza A vaccine, produced negligible antibody titers against wild type influenza virus indicating poor recognition of viral M2 and NP antigens respectively^12^. This result may have reflected the lack of an appropriate separation between the peptide epitopes^13^, and represented a setback for a universal flu vaccine based on these conserved peptide sequences^14^. Although the major immunodominant epitopes in HA are found within the strain specific HA head domain, most broadly neutralizing antibodies (bNAbs) against influenza virus have been shown to interact with the HA stem region through conserved conformational epitopes^15–18^, with a few of exceptions against the head domain^19,20^. Consequently, significant efforts to shift the focus of the antibody response from the head region to the conserved stem region have been made. For instance, elimination of the immunogenic head region of trimeric HA in an effort to focus the antibody response onto the conserved stem region has been performed^21^, while others have constructed chimeric head-stem HA variants^22^, or performed hyperglycosylation of the head domain in an attempt to mask its immunodominant behaviour^23^. On the other hand, depleting glycosylation on HA induced more cross-strain protection against influenza infections^24, 25^ likely resulted from exposing alternate immunogenic epitopes.

**Figure 1:**
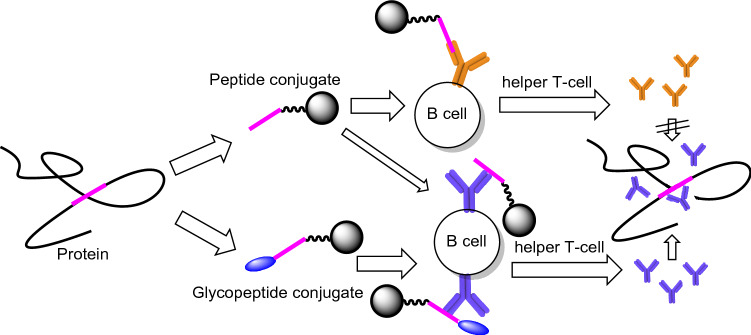
Glycosylation modulates the immune response towards peptide antigens. Peptide in a conjugate vaccine may act as a hapten to interact with specific B-cell receptors through its terminal neo-epitope, leading to antibodies that are not reactive to parental protein. On the other hand, a glycopeptide conjugate with this neo-epitope masked, favors the interaction of core amino acids with specific B-cell receptors, leading to the production of desired parental protein cross-reactive antibodies.

Carbohydrates are generally poor immunogens in comparison to proteins. The affinity of anti-carbohydrate antibodies is often observed to be 3–5 orders of magnitude lower relative to anti-protein or anti-peptide antibodies^26^. Furthermore, anti-glycan responses such as those directed to immunogenic polysaccharides^27,28^, rarely elicit antibodies directed to terminal saccharides on branch chains, due in part to these same structures being present in abundance on human glycoproteins; a phenomenon exploited by cancer cells to evade host immune responses^29^. Conjugation of saccharides to immunogenic proteins can render them more immunogenic however, it is often observed that these saccharide-protein conjugates produce significant responses against the linkage area^30^ as opposed to the saccharide; reinforcing their poor immunogenicity^31,32^. The relatively innocuous presence of terminal saccharides was exploited in the development of a pan-HA specific mAb for the quantification of multiple strains of influenza^33^. This study employed the use of a non-disclosed lactosylation technique as one of the measures to increase the solubility of an HA peptide for conjugation purposes, akin to phosphate modifications of HIV lipopeptides^34^. However, it was later found to be essential for the successful production of the HA peptide specific mAbs, and we speculated that this was potentially due to blocking and thus preventing the production of peptide terminal specific antibodies. Given that glycosylation can play a role in modulating glycoprotein immunogenicity, we hypothesized that masking one peptide terminus with a host carbohydrate molecule and conjugating the other terminus to a carrier protein, should serve to enhance peptide immunogenicity concomitant with redirecting antibody responses towards core peptide sequences that are in common with the homologous protein target (Fig. 1).

**Figure 2:**
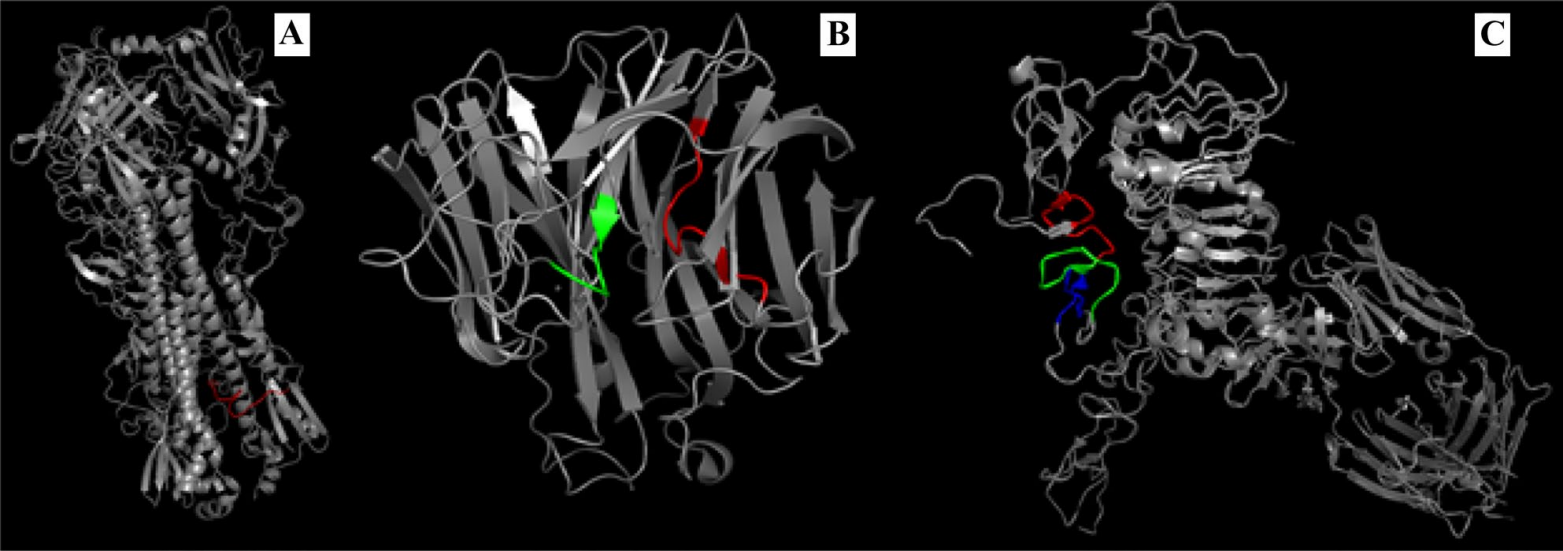
The peptide epitopes targeted in this report. (**A**) Influenza hemagglutinin: HA2 amino acids 1–14 (red); (**B**) influenza neuraminidase: amino acids 223–231 (red) and amino acids 291–296 (green); and (**C**) Human epidermal growth factor receptor 2: amino acids 270–281 (red), amino acids 282–294 (green) and amino acids 300–310 (blue). The epitope-highlighted structures were generated based on respective structures from https://www.rcsb.org/structure/5W6R (HA), https://www.rcsb.org/structure/1NN2 (NA), and https://www.rcsb.org/structure/3N85 (HER2).

In this communication, we provide evidence using immune responses to (glyco)peptide conjugates to confirm that glycosylation can indeed focus ensuing antibody responses to internal peptide core regions. The model peptides selected for study include a universal influenza viral sequence of a HA fusion peptide, NA peptides adjacent to its catalytic site, and peptides located within the critical dimerization loop of Her2 (Fig. 2).

## Results

### Immune response to HA (glyco)peptides

**Figure 3:**
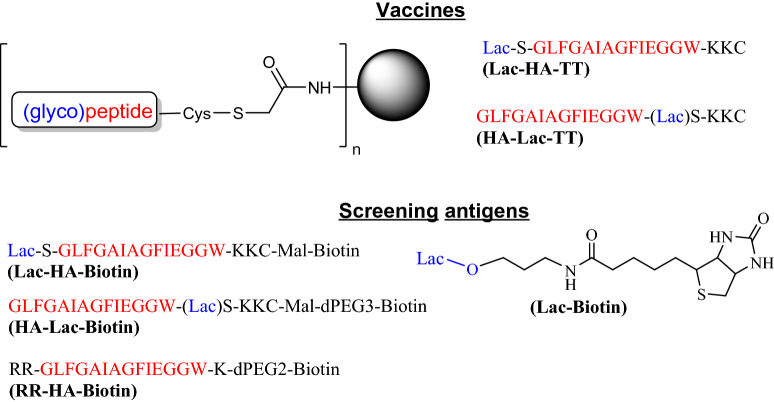
Influenza hemagglutinin fusion peptide-based conjugate vaccines and biotinylated screening antigens. Peptide conjugation to tetanus toxoid (TT) was monitored by SEC-HPLC on a Superose 6 10/300GL column. Parallel conjugations of both glycopeptides to BSA were also performed to ascertain peptide loading (c.a. 25% w/w) that was estimated by MALDI mass spectroscopic analysis. The use of parallel BSA conjugation with peptide constructs was to confirm peptide reactivity and relative loading levels that are difficult to quantify with larger protein constructs such as tetanus toxoid. For vaccine immunizations, we assumed that the TT conjugates have the same ratio of peptide/protein (w/w). Biotinylated coating antigens were prepared for screening, which include **Lac-HA-Biotin** and **HA-Lac-Biotin** (from homologous peptides), **RR-HA-Biotin** (extended by two arginines at the N-terminus), and **Lac-Biotin** to detect core peptide and lactose specific antibodies, respectively.

Previously, we have demonstrated that the KLH conjugate of a glycosylated HA peptide (a.a. 1–14 HA2) resulted in improved immunogenicity and specificity towards the peptide core sequence^33^. We reasoned these improvements arose from the effect of peptide glycosylation by (1) improvement on peptide solubility, and (2) masking the terminal neo-epitope present on the KLH conjugates. In order to test this hypothesis, we designed and synthesized two glycopeptide conjugates: one with lactose present at the peptide N-terminal (**Lac-HA-TT**) and the other with lactose intervening at the C-terminal conjugation site (**HA-Lac-TT**) (Fig. 3). The composition of these 2 peptide vaccine constructs was expected to shed light on both the solubility effect of lactose on immunogenicity, where both peptide constructs have similar solubility, and the influence of N-terminal lactose on diverting the production of irrelevant N-terminal binding antibodies to more relevant peptide core recognizing antibodies.

Antisera following three intranasal mouse immunizations were analysed by indirect ELISA to determine specific antibody titers (Table 1). The screening strategy employed biotinylated versions of the various peptide constructs to minimize carrier protein/linkage related antibody response detection. Four biotinylated peptide antigens synthesized included the vaccine homologous antigens (**Lac-HA-Biotin** and **HA-Lac-Biotin**) as well as **RR-HA-Biotin** with 2 additional arginines added at the N-terminus for detection of core specific antibodies, and **Lac-Biotin** for detection of lactose specific antibodies. Both **Lac-HA-TT** and **HA-Lac-TT** raised elevated levels of antibodies recognizing their respective antigens **Lac-HA-Biotin** and **HA-Lac-Biotin** (Titers > 12,800). **Lac-HA-TT** antiserum was able to also strongly recognize **HA-Lac-Biotin** (Titer ≥ 12,800) suggesting the induction of antibodies recognizing internal HA residues whereas **HA-Lac-TT** antiserum only minimally recognized **Lac-HA-Biotin** antigen where the free terminal residue is no longer present. Screening against **RR-HA-Biotin,** where again the terminal HA epitope is not present, demonstrated that only antibodies generated by **Lac-HA-TT** were capable of strong binding with only marginal recognition from **HA-Lac-TT** antibodies (Titer < 800). The poor binding by **HA-Lac-TT** antiserum to either **Lac-HA-Biotin** or **RR-HA-Biotin** suggests the presence of a significant amount of antibody specific to the terminal neo-epitope found in the immunogen conjugate. Furthermore, there was also no indication of lactose binding antibodies from either vaccine construct when screened against **Lac-Biotin** coating antigen. Overall, these results suggest that N-terminal glycosylation of HA peptides was able to focus antibody responses directed to the core region of the peptide epitope by masking the terminal peptide amino acids.

**Table 1:** Serum titer determination by indirect ELISA on immobilized HA biotinylated peptides and control.

Mouse	Immunized with	Post-immunization							
		Titer determined on							
		**Lac-HA-Biotin**		**HA-Lac-Biotin**		**RR-HA-Biotin**		**Lac-Biotin**	
		IgG1	IgG2a	IgG1	IgG2a	IgG1	IgG2a	IgG1	IgG2a
1	**Lac-HA-TT**	> 1:12,800	1:12,800	1:12,800	1:200	1:1600	1:100	< 1:100	< 1:100
2		> 1:12,800	1:6400	1:12,800	1:200	1:12,800	< 1:100	< 1:100	< 1:100
3		> 1:12,800	1:6400	> 1:12,800	1:800	> 1:12,800	1:400	< 1:100	< 1:100
4	**HA-Lac-TT**	1:6400	1:3200	1:12,800	1:6400	1:100	< 1:100	< 1:100	< 1:100
5		1:12,800	1:400	1:12,800	1:6400	1:800	< 1:100	< 1:100	< 1:100
6		1:6400	1:200	1:12,800	1:800	1:400	< 1:100	< 1:100	< 1:100

### Immune response to NA (glyco)peptides

**Figure 4:**
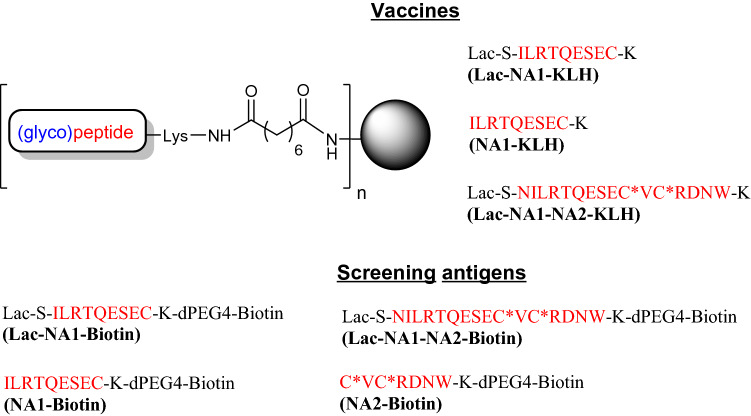
Influenza neuraminidase peptide-based conjugate vaccines and biotinylated screening antigens. The conjugate vaccines were synthesized through a bivalent linkage between lysine amino groups of (glyco)peptides and carrier protein, and purified by dialysis and centrifugal filters. The NA1 peptide containing a conserved amino acid sequence of 223–231 of neuraminidase was fused to a NA2 peptide (a.a. 291–296) which is a common epitope present in N1, 5, 7, 8, 9 influenza strains. A disulfide bridge (C*VC*) was introduced. Biotinylated (glyco)peptide screening antigens were prepared through derivatization of the C-terminal lysine amino group with the succinimidyl ester of dPEG4-biotin, and purified by HPLC on a C18-column.

Neuraminidase peptide NA1 (a.a. 223–231) is a conserved sequence found in multiple influenza strains^35^, and has been targeted for neuraminidase detection and quantification^36^. We employed a similar approach and made one peptide conjugate incorporating an N-terminal glycosylation (**Lac-NA1-KLH**) and the second lacking this modification (**NA1-KLH**) (see Fig. 4). Peptide conjugation to protein differed in terms of the carrier protein choice (KLH) and the use of a bivalent linker (disuccinimidyl suberate) to bridge peptide and carrier protein lysine residues. Following three intranasal mouse immunizations, the resulting mouse antisera were tested in ELISA using biotinylated versions of the two vaccine peptides (**Lac-NA1-biotin** and **NA1-biotin**). As expected, both peptide vaccines induced strong responses to their homologous peptide antigens (Titers >> 25,600, see Table 2). However, there were distinct differences in the antisera cross reactivity to the **Lac-NA1-biotin** and **NA1-biotin** screening antigens. The **Lac-NA1-KLH** vaccine raised significantly more antibodies capable of binding to **NA1-biotin** (Titer > 25,600) compared to the ability of **NA1-KLH** antiserum’s ability to recognize **Lac-NA1-biotin** (see Table 2). This result is consistent with our previous HA-TT conjugate observations, that suggested glycosylation decreases the immune response towards the terminal neo-epitope and correspondingly favours responses to the core peptide region. The results also suggest the unmodified peptide terminus in the peptide-conjugate functions like an immunodominant hapten producing antibodies less or not capable of binding to the core peptide sequence.

**Table 2:** Serum titer determination by indirect ELISA on immobilized NA1 biotinylated peptides.

Mouse	Immunized with	Post-2 immunization	
		Titer determined on	
		**Lac-NA1-Biotin**	**NA1-Biotin**
1	**Lac-NA1-KLH**	>> 25,600	> 25,600
2		>> 25,600	> 25,600
3		> 25,600	25,600
4		> 25,600	12,800
5	**NA1-KLH**	12,800	> 25,600
6		25,600	> 25,600
7		< 100	> 25,600
8		< 100	25,600

**Figure 5:**
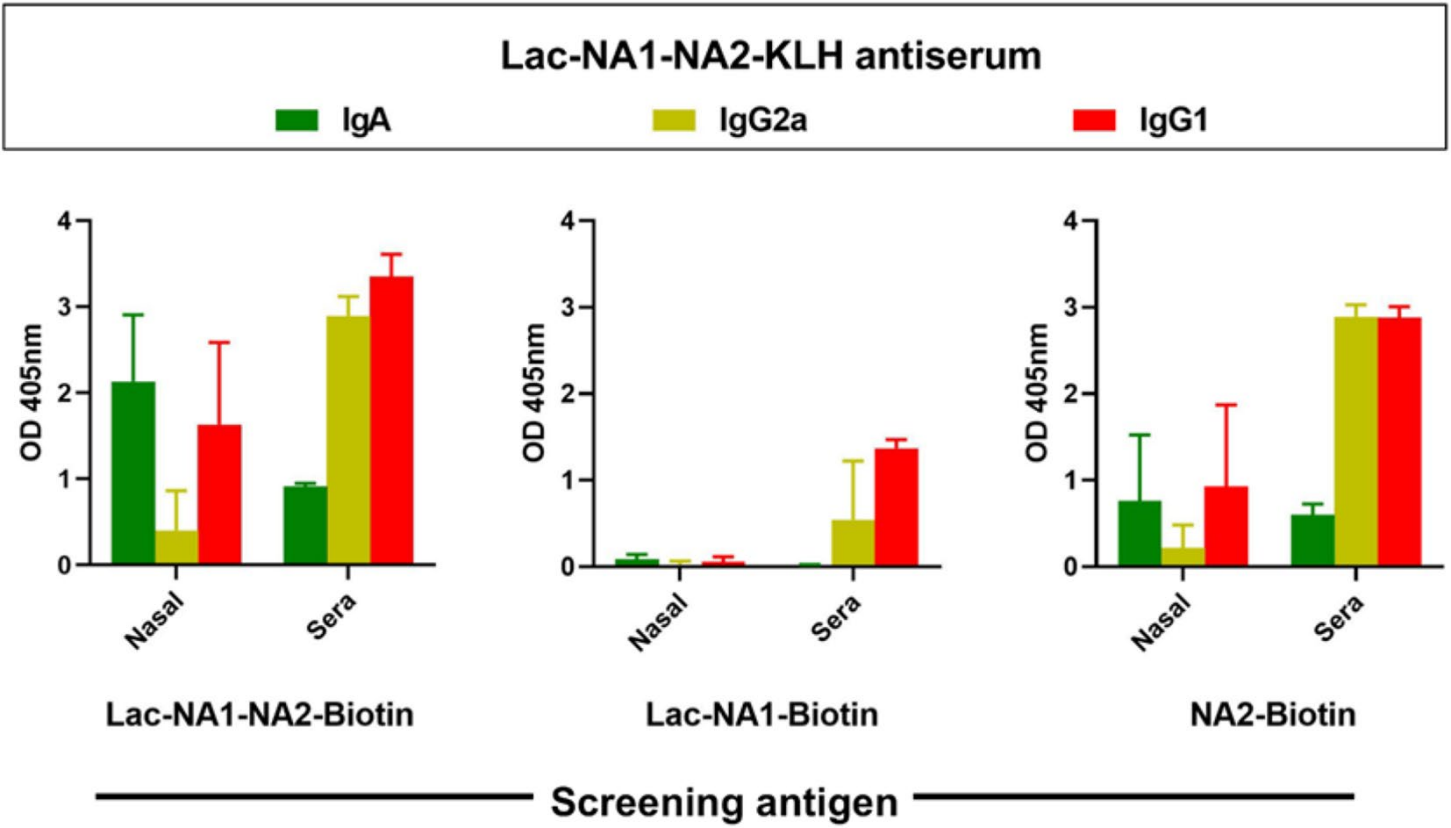
Epitope characterization of antiserum to an extended NA peptide glycoconjugate. The **Lac-NA1-NA2-KLH** construct was administered to mice (IN; n = 8) with cholera toxin adjuvant followed by re-administration on d15 and 22 and sera collection 14 days later. Binding profiles of pooled antisera were evaluated by ELISA using **Lac-NA1-NA2-biotin** (left), **Lac-NA1-biotin** (middle), and **NA2-biotin** (right) as coating antigens and the relative titers (OD_450_) expressed as the mean ± SD of triplicate wells.

To further investigate the masking effects of glycosylation on terminal peptide residues, we evaluated a longer NA peptide by fusing another relatively conserved **NA2** peptide (a.a. 291–296)^35^ to the **NA1** peptide yielding disulfide loop containing glycopeptide **Lac-NA1-NA2. Lac-NA1-NA2** was similarly conjugated to KLH (Fig. 4) and was intranasal administered to mice. The **Lac-NA1-NA2-KLH** conjugate induced both serum and mucosal IgA and IgG responses against its homologous **Lac-NA1-NA2**-**Biotin** antigen in indirect ELISA studies (Fig. 5). Interestingly, there was only poor recognition of the **Lac-NA1** component of the immunizing **Lac-NA1-NA2-KLH** construct, suggesting that the dominant antibody response was directed to the epitopes closer to the C-terminal, which was confirmed from the screening results employing the **NA2-Biotin** antigen (Fig. 5). This result suggests that the effect of glycosylation masking may be more relevant for nearby peptide residues and falls off with the distance from the sugar residues, although it can’t be ruled out that the epitope comprising NA2 was immunodominant.

### Immune response to Her2 (glyco)peptides

**Figure 6:**
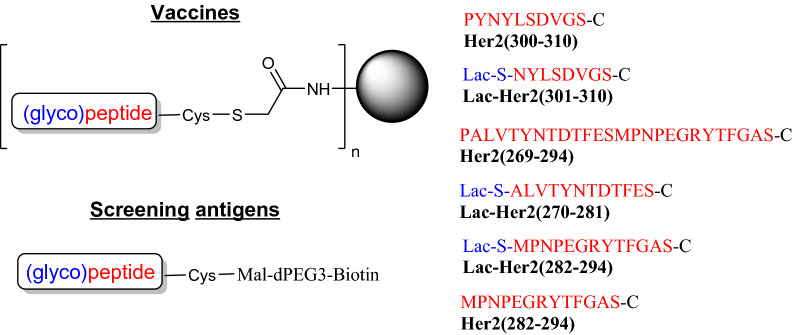
Target Her2 dimerization loop peptide-KLH and biotin conjugates. The conjugates and screening antigens were prepared through C-terminal cysteine and maleimide-KLH or maleimide-dPEG3-biotin respectively. Biotin conjugates were purified by HPLC on a C18-column.

**Figure 7:**
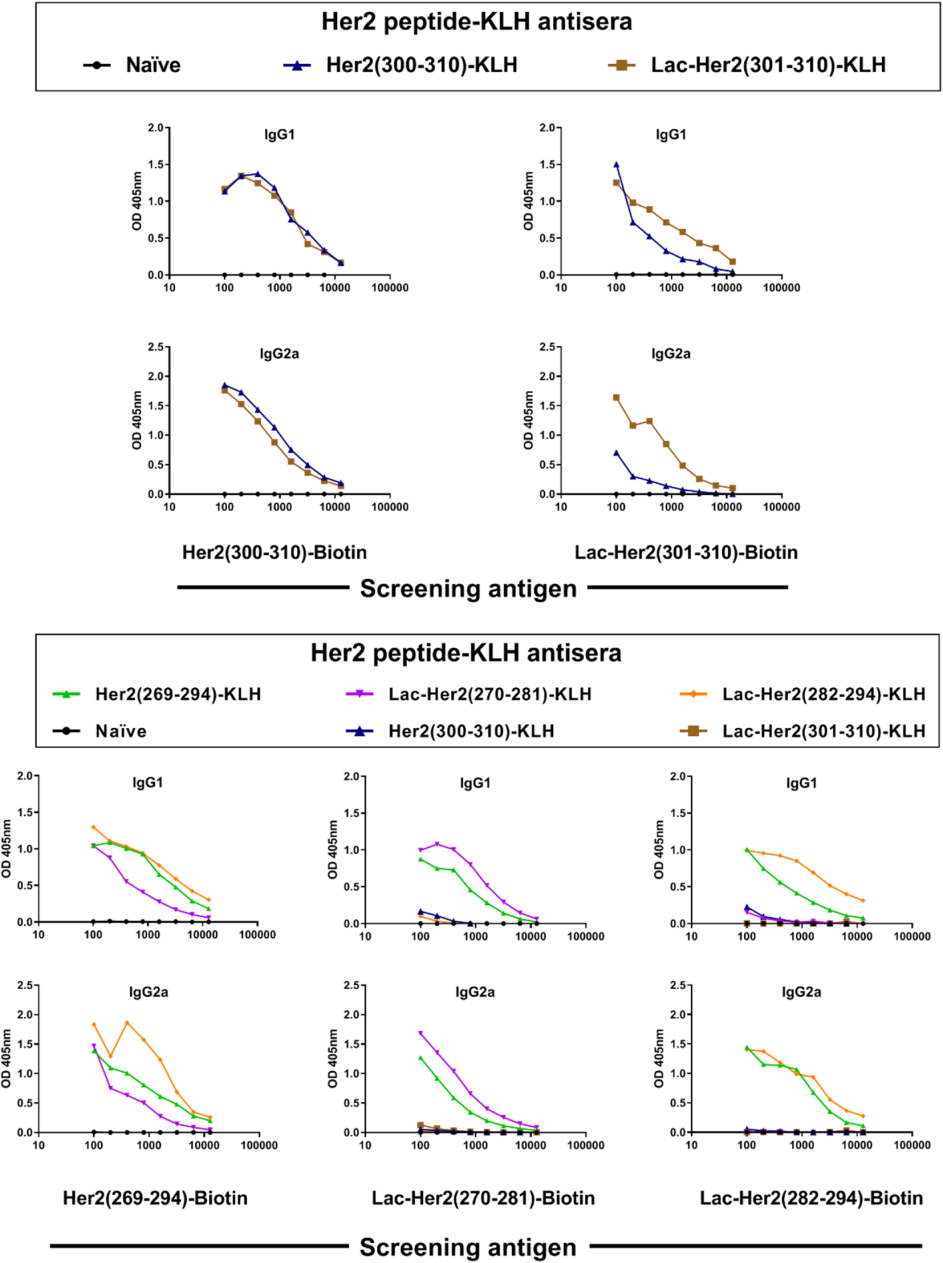
Illustrative binding curves from indirect ELISA analysis. Her2 peptide-KLH conjugates (listed in Fig. 6) in c-di-GMP adjuvant (10 μg) were injected subcutaneously into groups of female BALB/c mice (n = 5) on days 0, 14, and 21 and blood collected 14 days later. Indirect ELISA analysis on pooled antisera was performed on the indicated Her2-biotin screening antigens as previously described and isotype specific responses depicted.

Aberrant tyrosine kinase activity from ERBB2/Her2 receptor heterodimerization with other EGFR family members can lead to uncontrolled cell growth. A key region on Her2 precipitating this aberrant signalling is the dimerization loop (a.a. 266–333) that undergoes conformational changes upon heterodimerization^37^, and thus these peptide sequences are potential therapeutic targets to limit uncontrolled cell growth. Given this target, (glyco)peptides spanning a region of the dimerization loop (a.a. 269–310) were synthesized, conjugated to KLH, and mouse antisera were produced and evaluated (Fig. 6). The conjugates included unmodified Her2 peptides **Her2(269–294), Her2(282–294)** and **Her2(300–310)** and glycosylated peptides **Lac-Her2(270–281)**, **Lac-Her2(282–294)**, and **Lac-Her2(301–310)**. Reduced binding was observed for the un-glycosylated **Her2(300–310)-KLH** antiserum on the **Lac-Her2(301–310)-Biotin** coating antigen relative to the glycosylated **Lac-Her2(301–310)-KLH** antiserum, suggesting a proportion of the antibody response was specific to the terminal amino acid epitope (Fig. 7-top). This contrasted with the virtually equivalent binding profiles of **Lac-Her2(301–310)-KLH** and **Her2(300–310)-KLH** on the native **Her2-(300–310)** peptide antigen, supporting the premise that the majority of **Lac-Her2(301–310)-KLH** antibodies are specific to the internal amino acid residues of the peptide. This same disassociated binding profile was observed for other peptide conjugates (Fig. 7-bottom). The absence of cross-reactivity exhibited by the **Lac-Her2(270–281)-KLH** serum on **Lac-Her2(282–294)-Biotin** and by the **Lac-Her2(282–294)-KLH** serum on **Lac-Her2(270–281)-Biotin** further supported the non-immunogenic nature of the terminal lactose. Taken together, these results are entirely consistent with those obtained with HA and NA glycopeptides, and strongly supports the notion that the free terminal peptide regions of peptide vaccines can contribute significantly to the generation of non-productive antibodies that are not capable of recognizing parental proteins.

### Native Her2 recognition by (glyco)-peptide antisera

**Figure 8:**
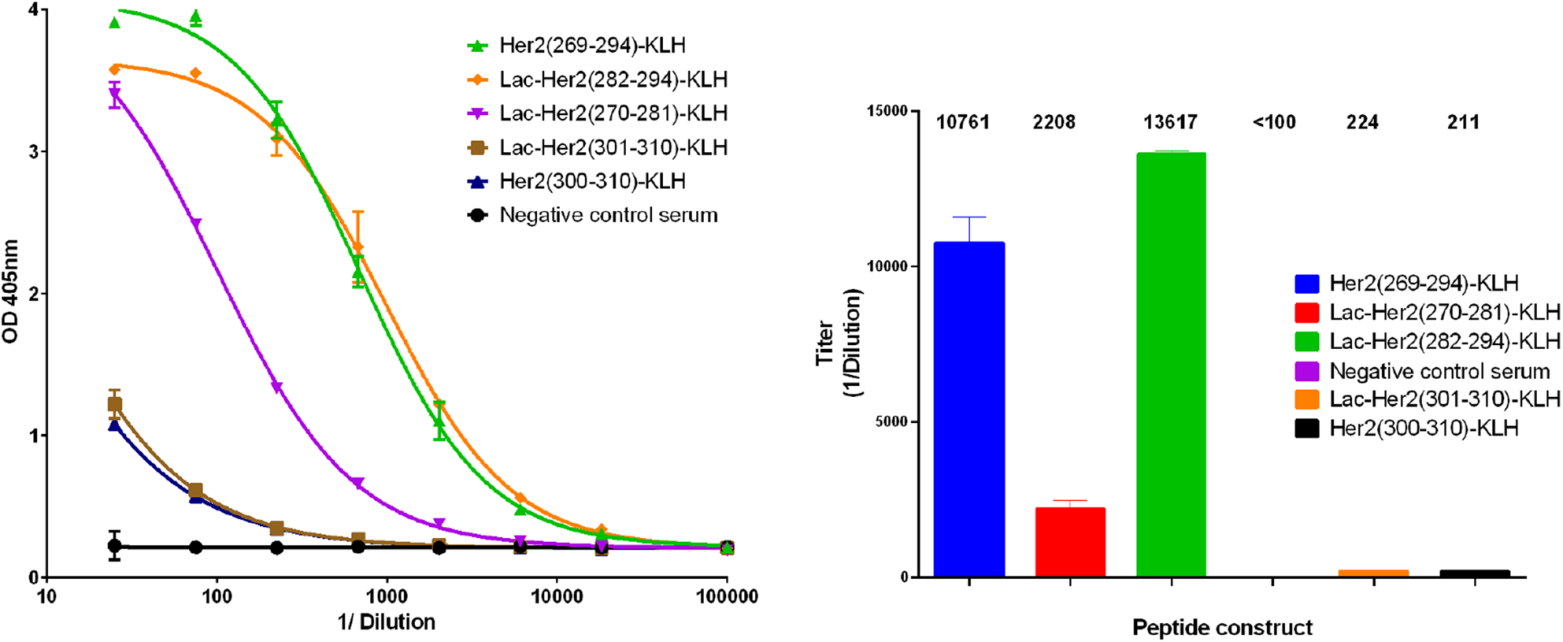
The binding to rHer2 by antisera. Indirect ELISA was performed as previously described using recombinant human Her2 protein as the screening antigen and the indicated Her2 peptide antisera or negative serum control. Absorbance values obtained at OD 405 nm using a multi-mode plate reader are plotted as the mean ± SD of triplicate wells (left panel) together with the corresponding endpoint titers ± SD (right panel). Data is representative of 3 individual experiments.

**Figure 9:**
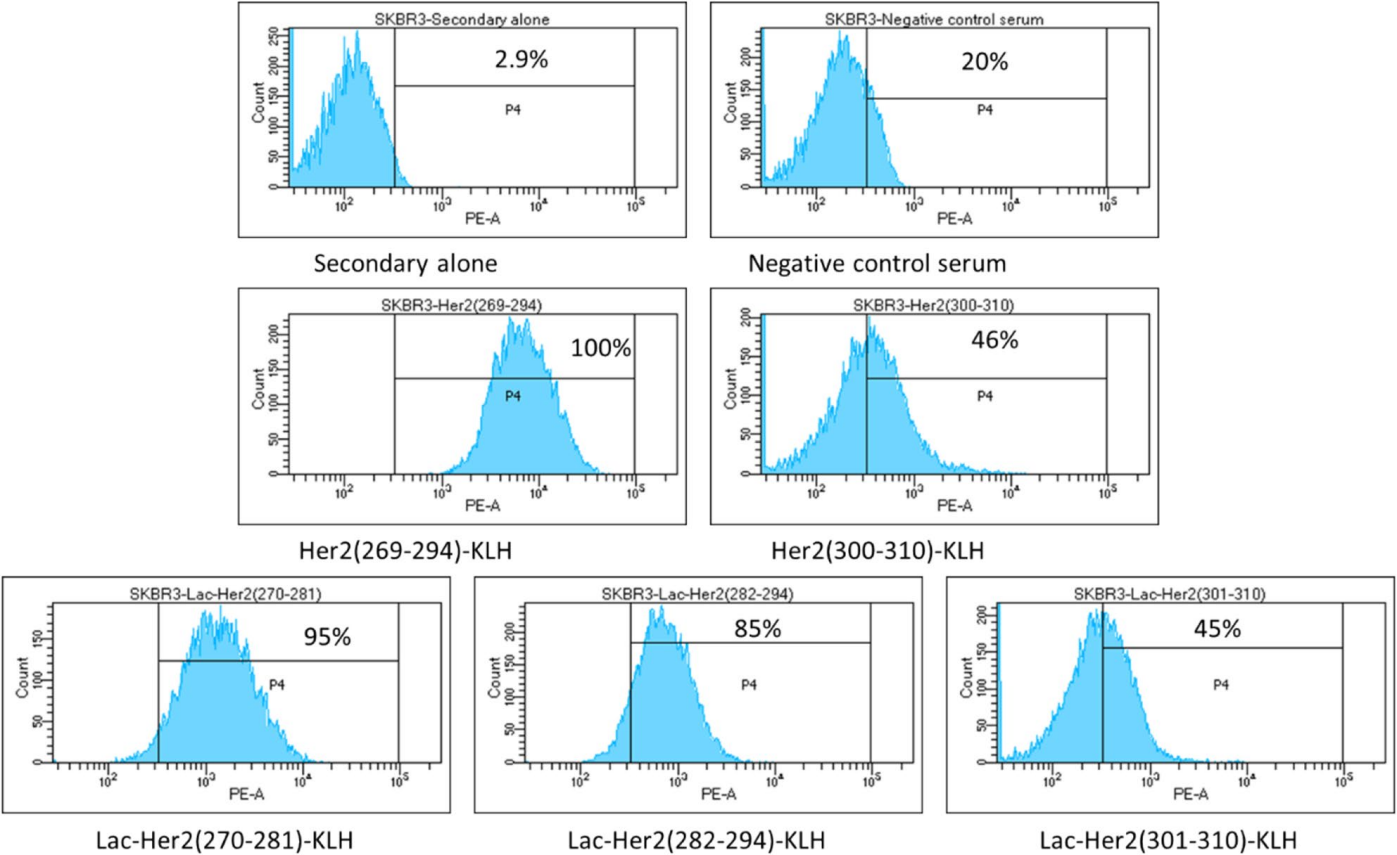
Recognition of cell surface Her-2 by antisera. Her2 expressing human breast carcinoma cells (SKBR3) were grown in RPMI-1640 media containing 10% FBS and harvested in log phase growth with trypsin/EDTA and washed with FACS staining buffer (1% BSA in PBS with 0.05% sodium azide). SKBR3 cells (0.5 × 10E6) were surface stained with a 1:10 dilution of the indicated mouse antisera (100 μl) for 30 min at 4 °C, followed by washing with staining buffer, and incubation for a further 30 min at 4 °C with goat anti-mouse IgG (H + L)-phycoerythrin. Negative controls consisted of naïve mouse serum and goat anti-mouse IgG (H + L)-PE secondary antibody alone. Binding data was acquired on a BDFortessa flow cytometer collecting 10 K live cell events, gating on the negative control, and the percent positive cells indicated in histograms. Data is representative of 2 individual experiments.

Examination of the binding of pooled antisera from 10 mice to recombinant Her2 protein was performed by indirect ELISA (Fig. 8). The antisera raised from **Lac-Her2(270–281)-KLH**, **Lac-Her2(282–294)-KLH**, and the long polypeptide **Her2(269–294)-KLH** were all capable of binding to recombinant human Her2 (Titers: 2207 ± 280, 13,617 ± 105, and 10,761 ± 825 respectively), whereas **Lac-Her2(301–310)-KLH** and **Her2(300–310)-KLH** antisera only marginally recognized rHer2 (Titers: 224 ± 4 and 211 ± 6 respectively). This latter result suggests the peptide sequence of amino acid 300–310 of Her2 is not accessible to antibody binding as opposed to the Her2 amino acid 269–294 sequence. The observed result that short glycopeptide conjugates can elicit antibody binding to full length rHer2 on par with a long Her2 polypeptide protein-conjugate (26 amino acids), confirms the recognition of whole protein sequences by antibodies raised to a minimal peptide immunogen that has had its terminal neo-epitope masked through glycosylation. To address whether the glycopeptide conjugate antisera is capable of recognizing Her2 in its cell membrane form, flow cytometry was performed using Her2 expressing human breast carcinoma cells (SKBR3) (Fig. 9). The antiserum from the long peptide conjugate **Her2(269–294)-KLH** not surprisingly possessed the strongest binding to SKBR3 cells (MFI = 6683) by virtue of the multiple B cell epitopes present within the 26 amino acid sequence. Interestingly, the **Lac-Her2(270–281)-KLH** antiserum, which bound plate immobilized rHer2 more weakly than the **Lac-Her2(282–294)-KLH** antiserum in ELISA studies, possessed more intense binding to cellular Her2 (MFI = 1460) relative to the **Lac-Her2(282–294)** antiserum (MFI = 700) at the single presumed saturating concentration of antisera evaluated. This unexpected cellular binding profile suggests the **Her2(282–294)** peptide epitope may be a more favourable target sequence due to potential better accessibility and/or flexible conformation of the cellular Her2 receptor.

### Functional activity of the glycopeptide antiserum on SKBR3 breast cancer carcinoma growth

**Figure 10:**
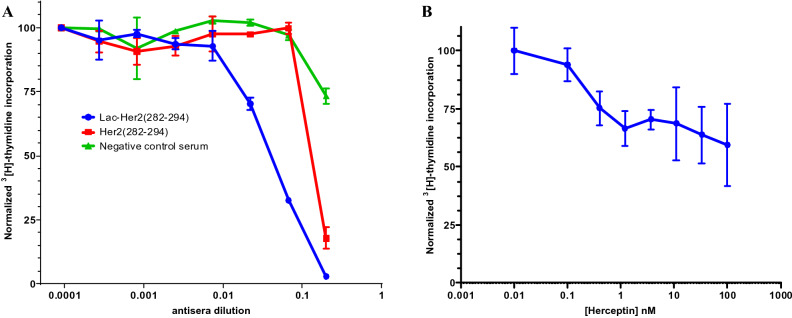
Growth inhibition of Her2 expressing human breast carcinoma cells (SKBR3) by antisera. (**A**) Mouse antisera to **Lac-Her2(282–294)-KLH**, **Her2(282–294)-KLH** or a **PBS negative control** were obtained as described previously. In a single experiment, triplicate wells of log phase growth SKBR3 cells were combined with serial threefold dilutions of **Lac-Her2(282–294)-KLH**, **Her2(282–294)-KLH**, **negative control serum** (**A**) or **Herceptin** (**B**) and were cultured in RPMI-1640 media containing 10% FBS for 48 h. During the last 18 h, 1 μCi/well of ^3^[H]-methyl thymidine was added, and harvested wells were enumerated for ^3^[H]-methyl thymidine incorporation on a Microbeta Trilux scintillation counter. The degree of proliferation was normalized to SKBR3 cells treated with media alone with error bars representing the mean ± SD.

To assess whether antiserum raised to a glycosylated Her2 peptide conjugate possessed functional activity, mouse immune sera was first generated to **Lac-Her2(282–294)** and the corresponding non-glycosylated **Her2(282–294)** peptides in a similar manner as described above. Indirect ELISA (Table 3) with pooled antisera (n = 10 mice) demonstrated an improved ability to recognize full length Her2 by the **Lac-Her2(284–294)-KLH** antiserum (Titer = 8823 ± 16) relative to the **Her2(284–294)-KLH** antiserum (Titer = 3731 ± 76); consistent with our previous observations on the effect of glycosylation to redirect the antibody response away from the terminal neo-epitope of the peptide vaccine. We next then employed an inhibition of SKBR3 proliferation assay using the antisera raised to **Lac-Her2(282–294)-KLH**, the corresponding non-glycosylated peptide antiserum from **Her2(282–294)-KLH**, and a PBS vehicle antiserum control (Fig. 10a). As a positive control, the therapeutic monoclonal antibody Herceptin was used at concentrations reported to inhibit proliferation by ~ 30% relative to untreated controls^38,39^. Following treatment of SKBR3 cells with dilutions of antisera or Herceptin, proliferation was measured after 48 h using ^3^[H]-thymidine uptake. As expected, Herceptin maximally inhibited SKBR3 proliferation in a dose dependent manner by ~ 40% relative to untreated SKBR3 cells (Fig. 10b). Significantly, **Lac-Her2(282–294)-KLH** antiserum was capable of completely inhibiting SKBR3 growth at high concentrations of antiserum, with the degree of inhibition diminishing in a dose dependant manner. The **Her2(282–294)-KLH** antiserum was clearly not as potent at growth inhibition relative to its glycosylated counterpart, suggesting a larger fraction of functional antibodies were present in the **Lac-Her2(282–294)-KLH** antiserum relative to the non-glycosylated **Her2(282–294)-KLH** antiserum, presumably due to the significant fraction of raised antibody that is specific to the N-terminal neo-epitope of the **Her2(282–294)-KLH** vaccine.

**Table 3:** Serum titer determination by indirect ELISA on native Her2 protein.

Pooled antisera (n = 15)	Immunized with		
	**Lac- Her2(282–294)- KLH**	**Her2(282–294)-KLH**	**PBS control**
Post-2 immunization titer on native Her2 protein	8823 ± 16	3731 ± 76	141 ± 1

## Discussion

This study further investigated observations from previous work using glycosylated peptide- protein conjugates to raise monoclonal antibodies for quantification of multiple strains of influenza^33^. During these studies, conserved HA peptide epitopes were lactosylated to increase peptide solubility but it was also observed to influence the epitope specificity of the raised antisera. A series of peptide specific mAbs were produced and a subset of 2 mAbs could be used as a universal reagent for detection and quantification of a large panel of influenza virus strains. Interestingly, only the lactose-peptide conjugate led to successful pan-HA mAb generation. Based on this unpublished observation, our strategy was to leverage the effects of terminal glycosylation to re-direct antibody responses to core internal peptide sequences as opposed to irrelevant terminal specificity. Based on previous experience with glycoconjugate vaccines, significant antibody responses to the linker region between the glycan and the carrier protein were often observed^30–32^. Taking advantage of this observation, we chose to replace the “linker” with a relevant peptide antigen such that the poorly immunogenic saccharides will function to mask the terminal peptide epitope potentially leading to productive immune responses to the peptide core epitope. This is particularly relevant when trying to generate effective parental protein antibody responses since the terminal peptide residue in the peptide-protein conjugate actually represents a neo-epitope that is not present in the target protein, and the presence of this neo-epitope can often dominate the ensuing antibody response. The method described in this report takes advantage of these glycan re-directing properties which we have now illustrated through several applications.

The conserved epitopes of influenza virus proteins have been highly desirable antigen targets for monoclonal antibody generation, with potential broad influenza therapeutic applications or as a universal virus quantification tool for seasonal vaccine production^40^. However, many attempts have failed to adequately raise specific antibodies to such universal peptide epitopes in mice using peptide fusion products^11^ or peptide conjugates^41–43^. Previously, broadly cross reactive rabbit anti-HA antisera were generated and were found to cross-react with all 16 subtypes of influenza A and 2 subtypes of influenza B tested^40^. However, the low titers obtained and the failure to raise antibodies in mice using the same peptide constructs, made the generation of mouse mAbs impracticable. The observed poor immunogenicity of the HA fusion peptide likely resulted from both the high hydrophobicity of the peptide and the presence of a terminal neo-epitope not found in HA protein, which may have behaved as an immunodominant epitope. Both factors could lead to undesired selection of B cell receptors leading to downstream production of antibodies irrelevant to the HA peptide epitope. Using the same HA fusion peptide (HA2, a.a. 1–14, GLFGAIAGFIEGGW) conserved across influenza A and B strains, we have shown that the addition of a poorly immunogenic and hydrophilic saccharide such as lactose at the peptide N-terminal position, improved its solubility and as shown in this study, efficiently blocks the terminal neo-epitope created within the synthesized peptide-conjugate immunogens. The effect of glycosylation on protein immunogenicity is not without precedence. For example, depletion of glycan on HA served to improve HA immunogenicity^24,25^, whereas enhanced glycosylation within the head region of HA diminished HA immunogenicity and resulted in an immune response redirected towards the stem region^23^. Therefore, modulation of a peptide or protein’s immunogenicity may be controllable through glycosylation strategies.

Incorporating lactose at either the N- or C-terminus of a 14-mer HA2 peptide conjugate vaccine (**Lac-HA-TT** or **HA-Lac-TT**) in combination with screening assays using different biotinylated peptide homologs (Table 1), allowed for several observation to be made. Importantly, these observations are independent of the specific characteristics of the immunizing constructs. The degree of peptide loading onto the various carrier proteins used in this study (TT and KLH) were difficult to quantitate. BSA peptide conjugate reference controls were synthesized in parallel where the extent of loading could be estimated by MALDI-MS. These controls provided us with: (1) a manner to qualify the activity of our peptide constructs for conjugation, and (2) a relative measure for estimating the degree of conjugation for immunization protocols. While we recognize the final degree of peptide loading onto TT or KLH may vary depending on the particular construct, and will need to be addressed in follow up studies, binding evaluation using a single peptide antiserum together with different screening antigens is agnostic in terms of the peptide-protein loading factor. In terms of intra-peptide conjugate comparisons, the similar profiles/titers observed with homologous peptide screening antigens suggests these differences may be minimal, however this caveat must be taken under consideration. From the differential screening with unmodified, glycosylated, or amino acid extended peptide antigens, the results strongly suggest that a sizeable proportion of antibodies generated to the **HA-Lac-TT** immunogen are directed towards the terminal neo-epitope of the peptide conjugate. In contrast, the glycosylated **Lac-HA-TT** conjugate reacted almost equivalently to HA antigen or HA antigens in which the terminal amino acid was blocked with either lactose or arginine residues, inferring the antibodies produced were primarily directed to the internal peptide sequences shared by all three screening antigens. Furthermore, the presence of the terminal neo-epitope appeared to be the major directing factor as opposed to peptide solubility since immunogens were constructed with the hydrophilic lactose located at either the N- or C-terminus of the peptide, with the latter construct behaving similarly to its non-glycosylated homolog. And lastly, there was no evidence that lactose was a determinant of any significant antibody response, further supporting the non-immunogenic nature and its use in glycopeptide vaccine conjugates.

We repeated this glycan-masking strategy using glycosylated derivatives of conserved NA peptides. Our results were in line with our previous observations using HA universal peptides and supported our hypothesis that the glycosylation effect can drive production of specific peptide core antibodies (Table 2). Interestingly, the effect of the glycosylation shift appears to fall off with distance from the glycosylation position, but also highlights the potential pitfalls of using linear peptide immunogens possessing multiple epitopes. For example, assuming the effective intervention target for flu infection is the peptide sequence **NA1** (Fig. 4) but the influenza vaccine incorporated the longer peptide structure **Lac-NA1-NA2**. Our results would indicate very little antibody to the desired **NA1** region would be generated since the alternate B cell epitope in this peptide was found to dominate in our observed immune response. Thus, the selection of a minimal peptide sequence for effector function can be critical and challenging, particularly when multiple epitopes are present and a non-functional epitope is immunodominant. The influence of our glycan re-directing strategy in this latter scenario may be minimal.

We have previously observed that mAbs specific to NA1 peptide, which were produced using a **Lac-NA1-KLH** conjugate, only weakly reacted with full length rNA (unpublished results). This was likely due to the fact that this epitope is located near the NA catalytic site where conformational restraint, accessibility, and steric hindrance exerted by neighbouring N-glycosylation may have all played a role^35^. Taken together, the experimental data from our HA and NA peptide studies clearly demonstrate the advantages but also some limitations of the method. It is clear that an accessible and flexible peptide target sequence has to be selected to maximize the probability of raising effective antibodies, and highly structured epitopes within α-helices and β-sheets or in regions of high protein glycosylation should be avoided. In this regard, application of our glycosylation induced antibody redirection approach for HA or NA therapeutics may not be practical, however accessible targets such as the dimerization loop of Her2 receptors may be an ideal target. This strategy may also find greater applicability in the development of diagnostic monoclonal antibodies such as has been demonstrated with our influenza strain quantification study.

The Her2 dimerization loop runs from amino acids 266 to 333 adjacent to rich disulfide regions and plays a key role in the formation of heterodimers with other EGFR family members such as EGFR1, 3, and 4 leading to elevated activation states and oncogenesis^37^. Apart from confirming the ability of N-terminal lactose to redirect antibody specificity towards core peptide regions consistent with our HA and NA studies, antisera raised to 2 short glycosylated peptide conjugates, **Lac-Her2(270–281)-KLH** and **Lac-Her2(282–294)-KLH**, were also capable of recognizing plate bound full length recombinant Her2 and importantly, native Her2 receptors on carcinoma cells. Binding recognition from these 2 antisera were at least on par with antiserum raised to a much longer Her2 peptide (269–294) conjugate incorporating multiple epitopes, although as discussed above, differences in conjugate construction may factor into this analysis. Effective responses against a short targeted Her2 peptide are more desirable than those elicited by longer polypeptides to prevent potential deleterious responses or the dominance of non-productive antibody responses to immunodominant epitopes. One of the proposed mechanisms for the Her2 specific therapeutic mAb, Herceptin, is cellular growth control as result of its binding to Her2 receptors^44^. We were able to demonstrate that the antibodies raised to the short glycosylated Her2 peptide, **Lac-Her2(282–294)**, were capable of inhibiting the growth of Her2 expressing SKBR3 carcinoma cells, and this growth inhibition effect was superior to what was observed with its non-glycosylated counterpart. Given the binding patterns of these two antisera, the greater cell growth inhibition of the **Lac-Her2(282–294)** antibodies is presumably due to better recognition of the cellular Her2 loop and interference of Her2 dimerization. In summary, glycosylation of a short relevant Her2 peptide induced functional antibodies to the short peptide core region, which subsequently were able to bind to and inhibit Her2 mediated SKBR3 breast carcinoma cell proliferation.

The selection of what linear peptide to target for vaccines, antibody therapy, or diagnostic tools requires an understanding of protein structure dynamics, particularly conformation changes associated with biological or immunological events. The more we know about this dynamic structural information, the more likely we will be able to effectively design and target the selected epitope. Although we have found that the masking effect on peptide epitopes can be exerted by various saccharides (e.g. lactose, glucose, and glucosamine, see Supplement Information), the exact impact of these modifications on productive immune responses has to be further defined in order to apply this technology in a predictive and productive manner. The findings of this study adds to our knowledge base and may be employed in the future for diagnostics, therapeutics, and possibly even vaccines.

In conclusion, we have demonstrated that (a) the immunogenicity of linear peptides, particularly hydrophobic peptides, can be improved by terminal glycosylation, (b) terminal glycosylation shifts the immune response away from irrelevant terminal neo-epitopes created in peptide conjugate vaccines and re-focuses this response towards the core peptide sequences, and (c) the antibodies targeting key core regions such as the dimerization loop of Her2 can be functional. Peptide target selection has to be made with knowledge of the dynamic changes that occur at the region of interest since static binding of antibody on immobilized proteins or virus may be mis-informative. Finally, this glycosylated linear peptide strategy is best suited to target key accessible regions and not regions constrained by higher order protein structures. Importantly, the application of this technology provides a pathway to raise specific antibodies to key, but otherwise non-immunogenic peptides, and could facilitate the development of therapeutic or diagnostic mAbs towards defined peptide epitopes.

## Material and methods

### Chemicals and reagents

Chemicals and reagents for conjugation were purchased from Sigma-Aldrich (Oakville, ON, Canada) unless otherwise stated, and used without further purification; peptides and glycopeptides were synthesized by Sussex Research Laboratories (Ottawa, ON, Canada). The carrier proteins were obtained from Sigma-Aldrich (KLH) and Statens Serum Institute, Copenhagen, Denmark (tetanus toxoid). Recombinant HA, NA and Her2 were purchased from SinoBiological (Burlington, ON, Canada).

### Peptide conjugation

The conjugates were prepared via a thio-ether bond between terminal Cys of the (glyco)peptide antigen and bromoacetyl KLH or TT or via an amide bond between terminal lysine amino group of (glycol)peptide antigen and the lysine amino groups on a carrier protein through a bivalent linker (disuccinimidyl suberate).

#### Protein activation

Protein (20 mg) dissolved in distilled water (2 mL) was added 10× PBS (2 mL), to above solution was added at room temperature bromoacetyl activated ester (9 mg) in 0.18 mL DMSO. The solution was kept at rt for 6 h, dialyzed against PBS (3 × 500 mL), and filtered through glass wool. KLH-Br was obtained in PBS (16.5 mL) c.a. 1 mg/mL. BSA-Br was obtained in PBS (20 mL) c.a. 1 mg/mL with 20–24 bromoacetyl group based on MALDI analysis (see Supplement Information).

#### Conjugation Method 1

To a solution of TT-Br, KLH-Br or BSA-Br/PBS (2 mL, 1 mg/ml PBS) was added (glyco)peptide antigen with terminal Cys in DMSO (1 mg in 0.2 mL DMSO), and TCEP (Mw 250.19) in PBS was also added (20 µL, 5 mg/mL). The solution was kept at room temperature overnight and the conjugation was confirmed by HPLC analysis. The conjugates were purified through Amicon tube (30 K), and purified conjugates were kept in PBS. The antigen loading was estimated by MALDI analysis (see Supplement Information).

#### Conjugation Method 2

(Glyco)peptide antigen with terminal Lys was reacted with excessive amount of disuccinimidyl suberate in DMSO, resulting in a monosuccinimidyl suberate (glycol)peptide, which after purification on HPLC was reacted with KLH and BSA in 10 X PBS overnight at room temperature to afford the desired conjugates (see Supplement Information).

#### Screening antigens

Biotinylated (glyco)peptides were prepared by coupling terminal Cys or Lys (amino group) to maleimide biotin or biotin activated ester respectively. Typically, a (glyco)peptide antigen was mixed with equivalent amount of biotin reagent in DMSO, incubated for 5 h at room temperature, diluted with water and lyophilized. The products were further purified on HPLC with a reverse phase C18 column with 0.1%TFA-water acetonitrile and lyophilized (see Supplement Information).

### Peptide conjugate analysis

Agilent Technology 1260 HPLC equipped with either a Superose-6 10/300 or a Superose-12 10/300 column with PBS as eluent was used for SEC-HPLC analysis of conjugation. Mass spectroscopic analysis were performed on SCIEX / Applied Biosystems 4800 TOF/TOF for MALDI-MS and on a Prince CE system (Prince Technologies, The Netherlands) coupled to a 4000 QTRAP mass spectrometer (AB Sciex, Canada) for ESI–MS, respectively. Protein concentration was estimated following procedures of a BCE kit.

### Animal immunizations

Female BALB/c mice were purchased from Charles Rivers Laboratories, St. Constant, Quebec, Canada and female A/J mice were purchased from The Jackson Laboratory, Bar Harbor, ME, USA. and housed in microisolators at the animal care facility at the National Research Council of Canada (Ottawa, Ontario, Canada). All studies were conducted in accordance with regulations and guidelines reviewed and approved by the Human Health Therapeutics Animal Care Committee and were conducted in facilities accredited by the Canadian Council on Animal Care.

#### Immunization with HA-(glyco)peptide conjugates

Groups of 3 six to eight-week-old female BALB/c mice were intranasally immunized with 100 µl (25 μg peptide antigen) of vaccine preparations or controls containing 1 μg of cholera toxin only at day 0, 15 and 22. At day 36, the mice were sacrificed, blood collected and serum prepared. Serum levels of specific IgG1 and IgG2a were measured by ELISA.

#### Immunization NA-(glyco)peptide conjugates

For NA1: groups of 4 six-week old female A/J mice were injected subcutaneously with 100 µg of conjugates emulsified in Titermax adjuvant at day 0 and 21. Blood was collected in microvette CB 300Z tubes at day 29, and serum was stored at − 20 °C until assay. For NA1-NA2: groups of 8 six to eight-week female BALB/c mice were intranasal immunized with 100 µl (5 μg peptide antigen) of vaccine preparations or controls containing 1 μg of cholera toxin only at day 0, 15 and 22. At day 36, the mice were sacrificed, blood collected and serum prepared. Serum levels of specific IgG1 and IgG2a were measured by ELISA.

#### Immunization with Her2-(glyco)peptide conjugates

Groups of 5 six to eight-week female female BALB/c mice were immunized subcutaneously with peptide conjugate vaccines (3 × 10 μg peptide antigen) with c-di-GMP adjuvant (10 µg) at day 0, 14 and 21. At day 35, the mice were sacrificed, blood collected and serum prepared. Serum levels of specific IgG1 and IgG2a were measured by ELISA.

### ELISA

High binding 96-well plates (Fisher Scientific, Ottawa, ON, Canada) were coated with 25 µL per well of NeutrAvidin at 10 µg/mL in 50 mM carbonate buffer at pH 9.8. After 2 h incubation, microplates were washed three times with PBS and blocked for 30 min with 1% BSA. Microplates were washed once with PBS and 25 µL of biotinylated peptides or biotinylated lactose at 5 µg/mL was added and incubated overnight at 4 °C. After 4 washes with PBS-Tween, 25 µL of serial dilutions of sera samples were added. After a 2 h incubation, microplates were washed 3 times with PBS-Tween and 25 µL of a 1/5000 dilution of alkaline phosphatase conjugated goat anti-mouse IgG (H + L) (Cedarlane Laboratories, Burlington, ON, Canada) in blocking buffer was added. After 1 h incubation, microplates were washed 4 times with PBS and 25 µL of p-nitrophenyl phosphate (pNPP) substrate at 1 mg/mL in carbonate buffer at pH 9.6 was added and further incubated for 30 min. Absorbance was read at 405 nm using a SpectraMax plate reader (Molecular Devices, San Jose, CA, USA).

### Flow cytometry

Her2 expressing human breast carcinoma cells (SKBR3) were grown in RPMI-1640 media containing 10% FBS (ThermoFisher Scientific, Ottawa, ON, Canada) and harvested in log phase growth with trypsin/EDTA and washed with FACS staining buffer (1% BSA in PBS with 0.05% sodium azide). SKBR3 cells (0.5 × 10E6) were surface stained with a 1:10 dilution of the indicated mouse antisera (100 μl) for 30 min at 4 °C, followed by washing with staining buffer, and incubation for a further 30 min at 4 °C with goat anti-mouse IgG (H + L)-phycoerythrin (Cedarlane, Burlington, ON, Canada). Gates were set on negative controls consisting of naïve mouse serum and goat anti-mouse IgG (H + L)-PE secondary antibody alone. Binding data (10 K live events) were acquired and analyzed on a BDFortessa flow cytometer (BD Biosciences, Mississauga, ON, Canada).

### Growth inhibition of Her2 expressing human breast carcinoma cells (SKBR3)

SKBR3 cells were grown in RPMI-1640 media containing 10% FBS (ThermoFisher Scientific, Ottawa, ON, Canada) and harvested in log phase growth with trypsin/EDTA. Triplicate wells of SKBR3 cells (20 K/ well) were combined with serial threefold dilutions of Lac-Her2(282–294)-KLH, Her2(282–294)-KLH, negative control serum or Herceptin (SinoBiological, Burlington, ON, Canada) and were cultured in RPMI-1640 media containing 10% FBS for 48 h. During the last 18 h, 1 μCi/well of ^3^[H]-methyl thymidine (Perkin-Elmer, Woodbridge, ON, Canada) was added, and harvested wells were enumerated for ^3^[H]-methyl thymidine incorporation on a Microbeta Trilux scintillation counter (Perkin Elmer). The degree of proliferation was normalized to SKBR3 cells treated with media alone.

## Supplementary information

Supplementary Information.
