# Supplementary material for: Masking terminal neo-epitopes of linear peptides through glycosylation favours immune responses towards core epitopes producing parental protein bound antibodies
Source: Sci Rep. 2020 Oct 28;10:18497. doi: 10.1038/s41598-020-75754-7 (PMC7595224; doi:10.1038/s41598-020-75754-7)
Supplement: Supplementary file 1 — Supplementary Information. [file 41598_2020_75754_MOESM1_ESM.doc]

**Supplemental information**

**Masking terminal neo-epitopes of linear peptides through glycosylation favours immune responses towards core epitopes producing parental protein bound antibodies**

Robert Pon, ^1^ Anne Marcil, ^1^ Wangxue Chen, ^1^ Christin Gadoury, ^1^ Dean Williams, ^1^ Kenneth Chan, ^1^ Hongyan Zhou, ^1^ Amalia Ponce, ^1^ Eric Paquet, ^2^ Komal Gurnani, ^1^ Anindita Chattopadhyay^1^ & Wei Zou, ^1^ *

*^1^ Human Health Therapeutics Research Center, National Research Council of Canada, 100 Sussex Drive, Ottawa, Ontario, Canada K1A 0R6*

*^2^ Digital Technology Research Center, National Research Council of Canada, 1200 Montreal Road, Ottawa, Ontario, Canada K1A 0R6*

**Table of contents**

1. Material and methods 3
2. Conjugate vaccines and coating antigens 3
3. Sugar masking effects on immunogenicity and antigenicity 8
4. **Material and methods**

*Material and reagents:* Chemicals and reagents for conjugation were purchased from Sigma-Aldrich and used without further purification; peptides and glycopeptides were synthesized exclusively by Sussex Research Laboratories, Ottawa, ON, Canada. Female BALB/c mice were purchased from Charles Rivers Laboratories, St. Constant, Quebec, Canada and female A/J mice were purchased from The Jackson Laboratory, Bar Harbor, ME, USA

*Analysis:* Agilent Technology 1260 HPLC equipped with either a Superose-6 10/300 or a Superose-12 10/300 column with PBS as eluent was used for SEC-HPLC analysis of conjugation. Mass spectroscopic analysis were performed on SCIEX / Applied Biosystems 4800 TOF/TOF for MALDI-MS and on a Prince CE system (Prince Technologies, The Netherlands) coupled to a 4000 QTRAP mass spectrometer (AB Sciex, Canada) for ESI-MS, respectively. Protein concentration was estimated following procedures of a BCE kit.

1. **Conjugate vaccines and coating antigens**

Conjugation: the conjugates were prepared via a thio-ether bond between terminal Cys of the (glyco)peptide antigen and bromoacetyl KLH or TT (see Figure 2a), or via an amide bond between terminal lysine amino group of (glycol)peptide antigen and the lysine amino groups on KLH or TT through a bivalent linker (disuccinimidyl suberate) (see Figure 1).

**SI-Figure 1.** Methods for (glyco)peptide-protein conjugation via terminal cysteine (a) or lysine (b).

*Protein Activation*: protein (20 mg) dissolved in distilled water (2mL) was added 10 x PBS (2 mL), to above solution was added at room temperature bromoacetyl activated ester (9 mg) in 0.18 mL DMSO. The solution was kept at room temperature for 6 h, dialyzed against PBS (3 x 500mL), and filtered through glass wool. KLH-Br was obtained in PBS (16.5 mL) c.a. 1mg/mL. BSA-Br was obtained in PBS (20 mL) c.a. 1 mg/mL (MALDI 68971-69448, 20-24, HPLC on Superose 6, elute: 0.4 mL PBS/min, retention: 41.755 min for BSA, 41.183 min for BSA-Br).

**Conjugation:**

*Method 1*: To a solution of TT-Br, KLH-Br or BSA-Br/PBS (2 mL, 1 mg/ml PBS) was added (glyco)peptide antigen with terminal Cys in DMSO (1 mg in 0.2 mL DMSO), and tris-(2-carboxyethylphosphin) hydrochloride (TCEP, Mw 286.65) in PBS was also added (20 µL, 5 mg/mL). The solution was kept at room temperature overnight and the conjugation was confirmed by HPLC analysis. The conjugates were purified through Amicon tube (30K), and purified conjugates were kept in PBS. The antigen loading was estimated by MALDI analysis (for results: see SI-Table 1).

*Method 2:* (glyco)peptide antigen with terminal Lys was reacted with excessive amount of disuccinimidyl suberate in DMSO, resulting in a monosuccinimidyl suberate (glycol)peptide, which after purification on HPLC was reacted with KLH and BSA in 10 X PBS overnight at room temperature to afford the desired conjugates.

**SI-**Table 1. Conjugates and estimated antigen loadings

| **Conjugate** | **Antigen loading (%)*** | **Conjugate** | **Antigen loading (%)*** |
| --- | --- | --- | --- |
| Lac-HA-TT | 25.0% | Her2(300-310)-KLH | 7.5% |
| HA-Lac-TT | 25.0% | Lac-Her2(301-310)-KLH | 10.0% |
| Lac-NA1-KLH | 25.0% | Her2(269-294)-KLH | 7.0% |
| NA1-KLH | 12.0% | Lac-Her2(270-281)-KLH | 15.0% |
| Lac-NA1-NA2-KLH | 16.0% | Lac-Her2(282-294)-KLH | 14.0% |
|  |  | Lac-Her2(282-294)-KLH | 15.0% (for experiment-Fig. 9) |
|  |  | Her2(282-294)-KLH | 11.0% (for experiment-Fig. 9) |

*Estimated based on their BSA conjugates

**The coating antigens:**

Biotinylated (glyco)peptides were prepared by coupling terminal Cys or Lys (amino group) to maleimide biotin or biotin activated ester, respectively. Typically, a (glyco)peptide antigen with terminal Cys was mixed with equivalent amount of biotin-maleimide, or a (glyco)peptide antigen with terminal amine (Lys) with equivalent amount of biotin N-hydroxysuccinimide ester in DMSO at room temperature and the solution was kept for 5 h. which was diluted with water and lyophilized to give product. The products were further purified on HPLC with a reverse phase C18 column with 0.1%TFA-water acetonitrile. The coating antigens for ELISA were obtained after lyophilization (see SI-Table 2).

**SI-**Table 2. Coating antigens and their structures

| Coating antigen | Structure | Calculated mass | Found mass |
| --- | --- | --- | --- |
| Lac-HA-Biotin |  | C_120_H_180_N_27_O_37_S_2_  2655.2 (2657.0) | 2688.8  (M + Na) |
| HA-Lac-Biotin |  | C_125_H_193_N_27_O_39_S_2_  2760.3 (2762.1) | 2761.4  (M + H) |
| RR-HA-Biotin |  | C_105_H_161_N_29_O_25_S  2260.2 (2261.7) | 2282.9  (M + Na) |
| Lac-Biotin |  | C_25_H_43_N_3_O_13_S  625.3 (625.7) | 626.4  (M + H) |
| Lac-NA1-Biotin* |  | C_87_H_150_N_20_O_37_S_2_  2131.0 (2132.4) | 2132.7  (M + H) |
| NA1-Biotin* |  | C_72_H_125_N_19_O_25_S_2_  1719.9 (1721.0) | 1720.7  (M + H) |
| Lac-NA1-NA2-Biotin |  | C_124_H_201_N_33_O_48_S_3_  3016.3 (3018.3) | 3017.8  (M + H) |
| NA2-Biotin |  | C_65_H_102_N_18_O_19_S_3_  1534.7 (1535.8) | 1536.0  (M + H) |
| Her2(300-310)-Biotin |  | C_86_H_128_N_18_O_30_S_2_  1956.8 (1958.2) | 1979.7  (M + Na) |
| Lac-Her2(301-310)-Biotin |  | C_96_H_147_N_19_O_40_S_2_  2269.9 (2271.4) | 2292.8 (M + Na) |
| Her2(269-294)-Biotin |  | C_159_H_235_N_37_O_53_S_3_  3606.6 (3609.0) | 3607.5  (M + H) |
| Lac-Her2(270-281)-Biotin |  | C_107_H_165_N_21_O_44_S_2_  2512.1 (2513.7) | 2534.9  (M + Na) |
| Lac-Her2(282-294)-Biotin |  | C_109_H_167_N_25_O_41_S_3_  2578.1 (2579.8) | 2578.9  (M + H) |

1. **Sugar masking effects on immunogenicity and antigenicity**

Conjugates

In order to investigate if the masking effect can also be exerted by other sugars or whether this effect is similar with C-terminal glycosylation, we prepared four peptide-KLH conjugates (see SI-Figure 2) using method b as illustrated in SI-Figure 1, with lactose, glucose and N-acetyl glucosamine O-glycosylated at C-terminal serine. The antigen loading was also estimated based on their BSA conjugates and presented as w/w percentages which were in the range of c.a. 15-20%.

**SI-Figure 2**. Peptide-KLH conjugates with masking sugar placed at the C-terminal. The percentage (w/w) of peptide in the conjugates are shown in the blanket.

Vaccination

6-8 week female BALB/c mice (n = 8) were immunized with 5 µg peptide and 1 µg cholera toxin for each intranasal immunization, and 5 mice were used in control group. The second and third immunization were performed at day 14 and 21, and at day 35 mice were killed, blood for serum were collected. Antigen-specific IgA, IgG1 and IgG2a were analyzed by ELISA.

Antisera from four conjugate vaccines were screened against two coating antigens, Lac-NA1-NA2-Biotin and NA1-Biotin, previously prepared. These antigens have epitope overlaps, which are also found in the peptide conjugate sequence. Vaccines, either with or without a terminal sugar, raised antibodies reactive to NA1-Biotin, though the titers varied (see SI-Figure 3). It appears that glycosylation can mask the peptide and lead immune response toward epitopes away from the glycosylation site, on the other hand, glycosylation (Glc and GlcNAc) may also make short peptide less immunogenic. The fact that those antisera were non-reactive to Lac-NA1-NA2-Biotin indicates that the antigenic epitope in the coating antigen can also be masked by glycosylation. Furthermore, it is not surprising that the antisera from KLH-Pep were not reactive against Lac-NA1-NA2-Biotin. The results were indeed in agreement with other observations in this study, confirming that two populations of antibodies, one against the core, and the other against the irrelevant terminal amino acid, were produced. It is plausible that the reaction of core-specific antibodies to the antigenic epitope in Lac-NA1-NA2-Biotin was likely blocked by the masking effect of lactose.

Apparently, more studies are needed in order to take advantage of the peptide glycosylation to better design epitope-based vaccine.


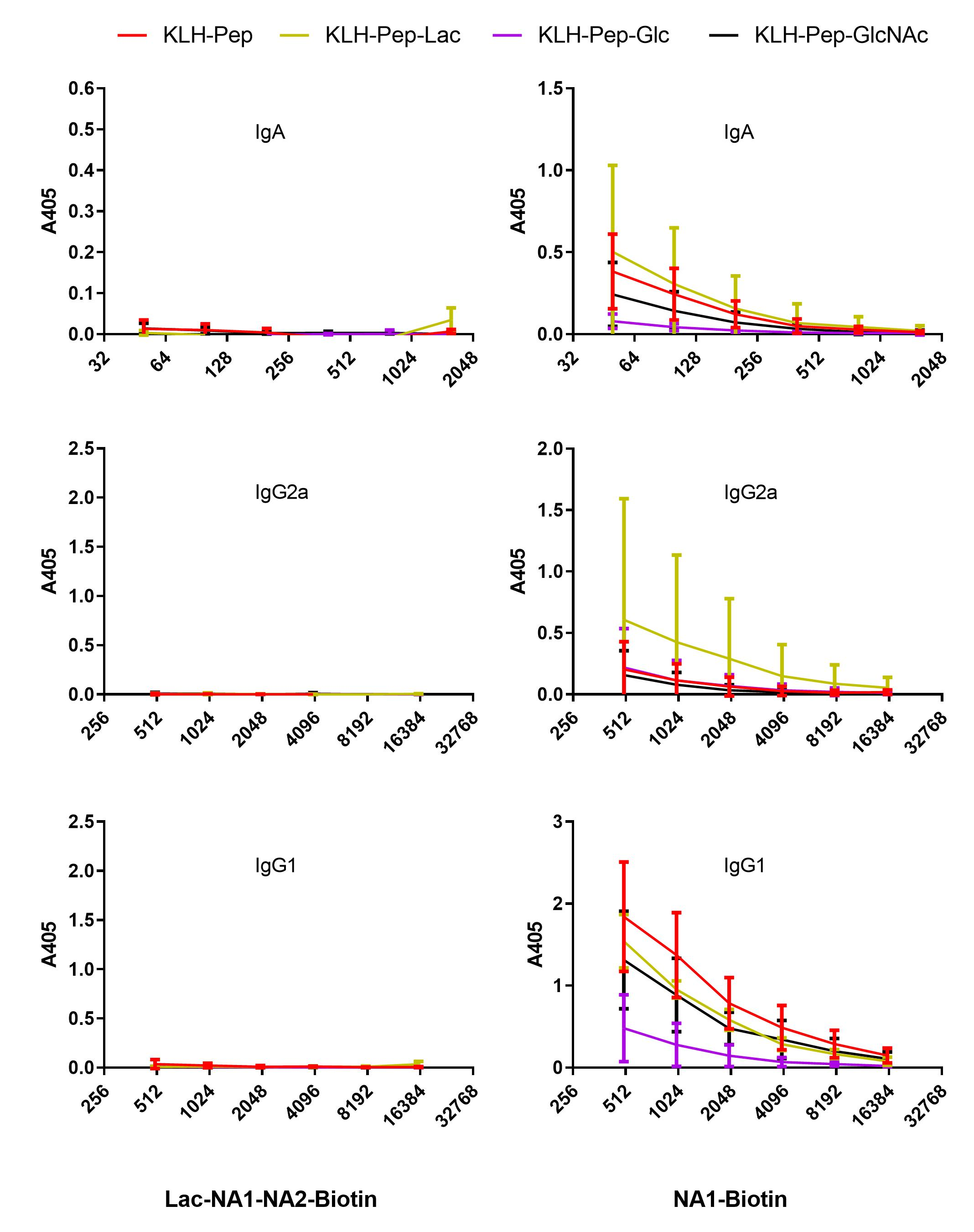


**Si-Figure 3. Indirect ELISA binding profiles.** N-terminus linked peptide-KLH conjugates produced antibodies in mice that recognized an antigenic epitope in a C-terminus linked NA1-Biotin, but not a glycosylated Lac-NA1-NA2-Biotin due to antigenic epitope masked by glycosylation. ELISA coating antigens (Lac-NA1-NA2-Biotin and NA1-Biotin) were coated in wells of Pierce™ NeutrAvidin™ Coated 96-well Plates by following manufactory’s instruction. An initial 1:50 serum dilutions were made on individual samples, followed with 1:2 series of dilutions, and assayed for IgA against the two coating antigens. For assaying IgG1 and IgG2a, initial serum dilution is 1:500, then followed with 1:2 series of dilutions. Alkaline phosphatase – conjugated -goat anti-mouse IgA, IgG2a and IgG1 and p-nitrophenylphosphate subtrate were used for the ELISA assay. Absorbance values were obtained at OD 405 nm using a multi-mode plate reader.
